# Copper binding alters the core structure of amyloid fibrils formed by Y145Stop human prion protein†

**DOI:** 10.1039/d4cp03593c

**Published:** 2024-10-08

**Authors:** Vidhyalakshmi Sridharan, Tara George, Daniel W. Conroy, Zach Shaffer, Witold K. Surewicz, Christopher P. Jaroniec

**Affiliations:** a Department of Chemistry and Biochemistry, The Ohio State University Columbus OH USA jaroniec.1@osu.edu; b Department of Physiology and Biophysics, Case Western Reserve University Cleveland OH USA

## Abstract

Transmissible spongiform encephalopathies (or prion diseases) such as Creutzfeldt-Jacob disease, mad cow disease, and scrapie are characterized by accumulation in the brain of misfolded prion protein aggregates (PrP^Sc^) that have properties of amyloid fibrils. Given that transition metal ions, such as copper and zinc, appear to be important for physiological functions of cellular PrP (PrP^C^) as well as for prion disease pathogenesis, exploring their role in the protein aggregation process is of considerable interest. Copper(ii) in particular is well-known to bind to the four tandem octapeptide repeats (PHGGGWGQ) located in the N-terminal region of PrP (human PrP amino acids 60–91), as well as to additional histidine binding sites outside the octarepeat region with distinct binding modes depending on Cu^2+^ concentration. Here, using the Y145Stop human prion protein variant (huPrP23-144) as a model and a combination of multidimensional solution and solid-state NMR spectroscopy, atomic force microscopy and thioflavin T fluorescence assays we probed the binding of Cu^2+^ to monomeric huPrP23-144 and the impact of this binding on fibril assembly kinetics and their structural properties. Remarkably, we found that fibrils formed by huPrP23-144 containing one molar equivalent of bound Cu^2+^ adopt a core structure that is distinct from that found for huPrP23-144 in the absence of Cu^2+^ but, instead, corresponds to a conformational strain formed by huPrP23-144 containing the A117V mutation. A similar huPrP23-144 A117V-like amyloid core structure was adopted by a Cu^2+^-bound Δ51-91 huPrP23-144 deletion variant lacking the entire octarepeat region, suggesting that Cu^2+^ binding to His residues 96, 111 and 140 located near the C-terminus of huPrP23-144 is primarily responsible for the observed change in fibril conformation, potentially due to partial structuring of the intrinsically disordered huPrP23-144 by the bound Cu^2+^ during the fibril assembly process. We also found that fibrils formed by Cu^2+^-bound huPrP23-144 adopt the native huPrP23-144-like rather than A117V-like structure when the fibrillization reaction is seeded with pre-formed huPrP23-144 amyloid.

## Abstract 2

Binding of copper(ii) to Y145Stop human prion protein leads to formation of an amyloid structural strain that is distinct from that obtained for amyloid fibrils of the same protein formed in the absence of bound copper(ii).

## Graphical abstract

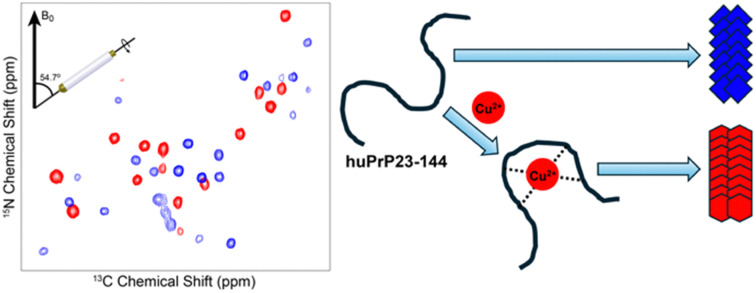

## 1. Introduction

Misfolding of proteins from their native states into β-sheet rich amyloids under physiological conditions is associated with a number of neurodegenerative diseases.^1^ One class of these disorders are transmissible spongiform encephalopathies (or prion diseases) which include Creutzfeldt–Jakob disease in humans, scrapie in sheep, and mad cow disease in cattle. The main pathogenic event in all these disorders is the conformational conversion of the cellular prion protein (PrP^C^) into infectious “scrapie” isoform (PrP^Sc^) that has properties of amyloid fibrils.^2–5^

It is well-established that, akin to several other proteins involved in neurodegenerative diseases,^6,7^ prion protein both *in vitro*^8^ and *in vivo*^9^ binds copper(ii) and other transition metal ions. Therefore, there is significant interest in detailed understanding of the mechanism of this interaction and its role in normal function of PrP^C^ as well as in disease pathogenesis. In a series of previous studies, Cu^2+^ binding to PrP has been investigated *in vitro* using a variety of biophysical techniques, including electron paramagnetic resonance (EPR) and solution nuclear magnetic resonance (NMR).^6,10–14^ Collectively, these studies have demonstrated that the four tandem octapeptide repeats PHGGGWGQ within the N-terminal part of the protein [residues 60-91 in human PrP (huPrP)] can bind up to four equivalents of Cu^2+^, and that histidine residues outside of the octarepeat region (H96, H111 and H140) serve as additional Cu^2+^ binding sites. Moreover, at sub-stoichiometric concentrations, Cu^2+^ ions coordinate to multiple histidine side-chains with a sub-nanomolar affinity.

Copper(ii) binding to PrP^C^ has been suggested to play a role in PrP^C^ trafficking and endocytosis, synaptic adhesion and signalling, and brain metal homeostasis.^13,15,16^ Even more important, it was found that Cu^2+^ ions can modulate PrP amyloid formation *in vitro*,^17–21^ and that binding of this metal ion correlates with formation *in vivo* of structurally distinct PrP^Sc^ strains associated with two different subtypes of sporadic Creutzfeldt–Jakob disease.^22^

To gain molecular level insight into the mechanism by which binding of Cu^2+^ ions may lead to formation of structurally distinct PrP amyloid strains, we focus here on fibrils formed by the C-terminally truncated Y145Stop human PrP variant (huPrP23-144) associated with a familial prionopathy.^23^ This PrP variant has been previously extensively used as a model for investigating *in vitro* formation of distinct prion amyloid strains and the phenomenon of seeding barriers.^24^ In particular, our previous solid-state NMR and cryo-EM studies of huPrP23-144 amyloid fibrils have established that: (i) fibrils contain a relatively rigid parallel-in-register β-core region spanning residues ∼112–141 and a dynamically disordered ∼90-residue N-terminal domain,^25–29^ (ii) deletion of most of the N-terminal domain, up to residue ∼98, results in fibrils with a wild-type (WT) core structure and assembly kinetics,^30^ and (iii) mutations and deletions of certain residues within the amyloid core region can alter the kinetics of amyloid assembly and/or protein conformation within the fibril core.^27,31–34^

In the present study, we used a combination of multidimensional magic-angle spinning (MAS) solid-state NMR—a technique that has previously been successfully applied toward structural studies of other amyloid-metal ion complexes^35–40^—with solution NMR, atomic force microscopy (AFM) and thioflavin T (ThT) fluorescence to investigate Cu^2+^ binding to huPrP23-144 and its impact on the amyloid core conformation and fibril assembly kinetics. Additionally, since, akin to full-length prion protein, huPrP23-144 contains multiple Cu^2+^ binding sites, we have assessed the relative importance of Cu^2+^ binding to these different sites by performing similar studies with the Δ51-91 huPrP23-144 deletion variant that, despite lacking the entire octarepeat region, adopts the WT huPrP23-144 conformation in the amyloid state.^30^

## 2 Results and discussion

### 2.1 Cu^2+^ binding to monomeric huPrP23-144 in solution

**Fig. 1:**
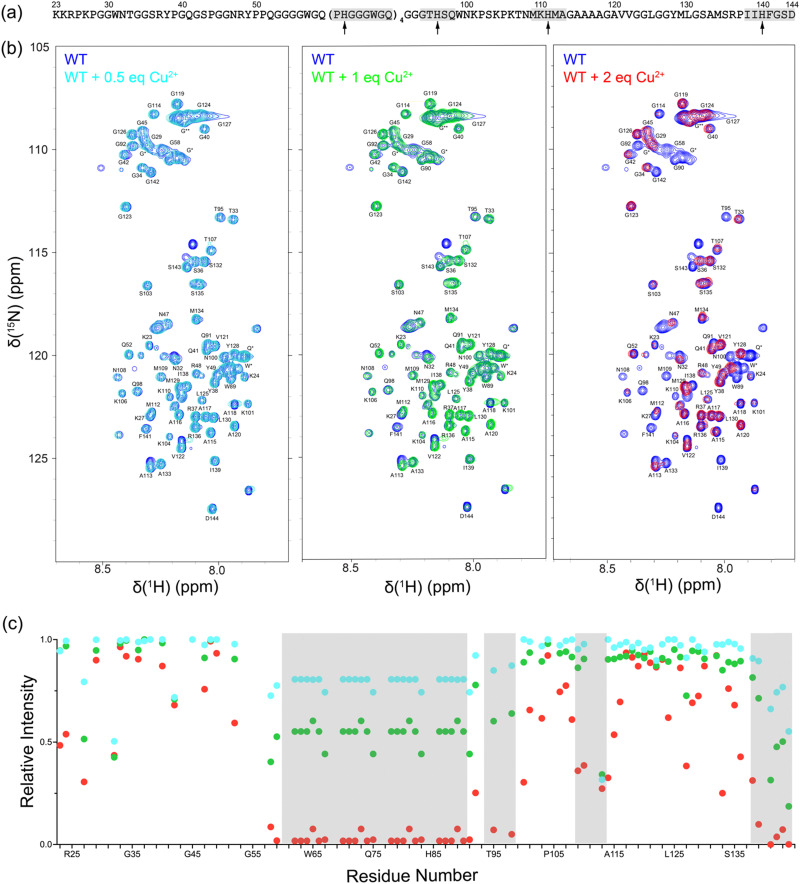
(a) Amino acid sequence of huPrP23-144. The locations of histidine residues are indicated by arrows and regions around these histidines showing the highest Cu^2+^ binding propensity and most pronounced reduction in resonance intensity due to paramagnetic relaxation enhancement [*cf.*, panel (c)], including the 60–91 segment that contains the tandem octapeptide repeats and regions around H96, H111 and H140 are highlighted in gray rectangles. (b) 850 MHz 2D ^15^N–^1^H HSQC solution NMR spectra of monomeric huPrP23-144 in the presence of 0.5, 1 and 2 molar equivalents of Cu^2+^ as indicated in the insets. Overlapping resonances marked with * and ** correspond to octarepeat region residues, with ** denoting glycine residues preceded by other glycines. (c) Peak intensities for huPrP23-144 residues in the presence of 0.5 (cyan), 1 (green) and 2 (red) molar equivalents of Cu^2+^ relative to the corresponding intensities in the absence of Cu^2+^ as a function of residue number. The gray rectangles correspond to regions with the highest Cu^2+^ binding propensity highlighted in panel (a).

First, we investigated Cu^2+^ binding to monomeric, intrinsically disordered huPrP23-144 by using solution NMR spectroscopy. Fig. 1 shows 2D ^15^N–^1^H heteronuclear single quantum coherence (HSQC) NMR spectra of huPrP23-144 recorded in the presence of increasing amounts of Cu^2+^ in the 0.5–2 molar equivalent range. These spectra clearly demonstrate Cu^2+^ concentration-dependent decrease in cross-peak intensity for multiple residues, primarily those located in the octarepeat region and in the vicinity of histidines 96, 111 and 140, due to paramagnetic relaxation enhancement.^41^ This is indicative of Cu^2+^ binding at all of these sites and consistent with earlier reports regarding high-affinity interactions between Cu^2+^ ions and multiple histidine residues in PrP.^6,10–14^ Given that at sub-stoichiometric Cu^2+^ concentrations the Cu^2+^ ions coordinate to multiple histidine side-chains and that paramagnetic relaxation enhancements for backbone ^1^H^*N*^ nuclei depend strongly on several factors including ^1^H^*N*^–Cu^2+^ distance and local protein dynamics, we did not attempt to correlate the differences in peak intensities for the individual residues with Cu^2+^ binding affinities of the different histidine sites.

### 2.2. Cu^2+^-bound huPrP23-144 fibril formation kinetics and morphology

**Fig. 2:**
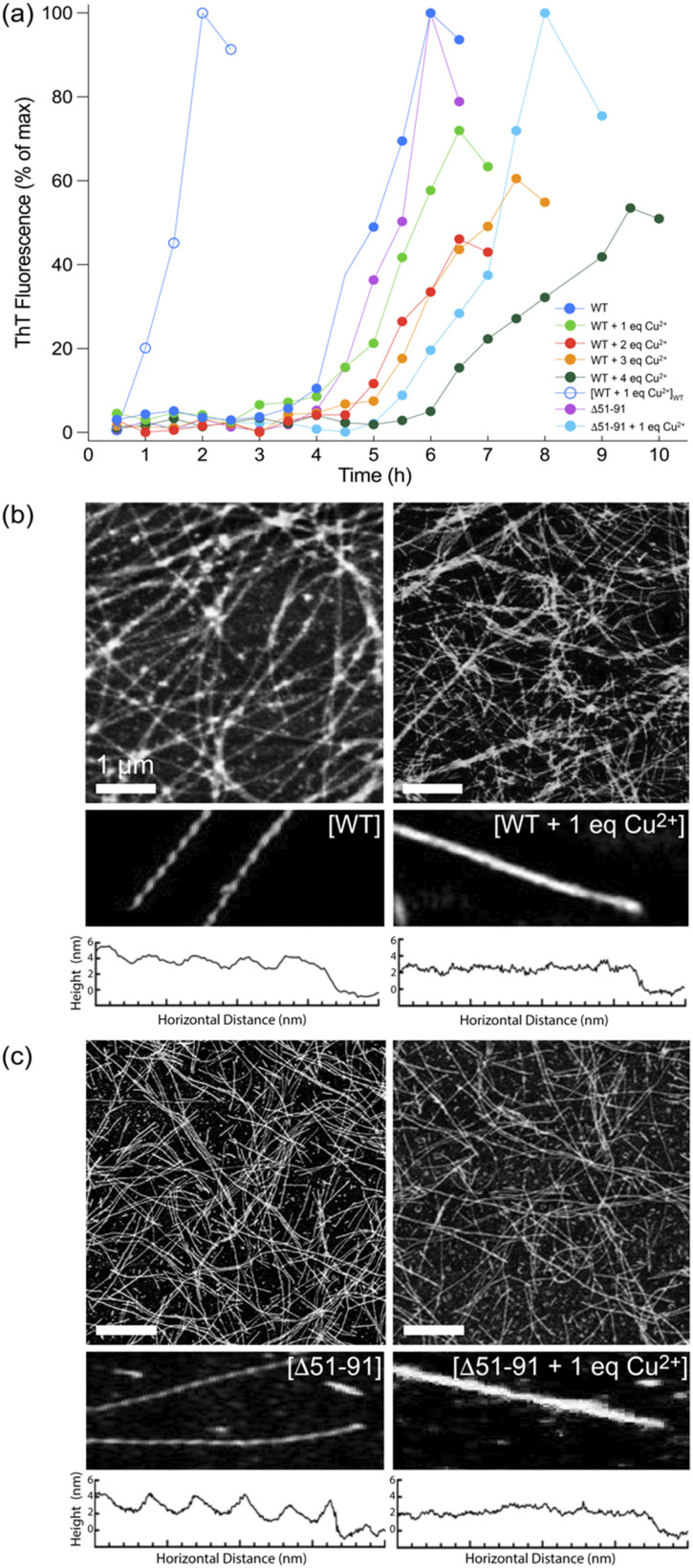
(a) Kinetics of amyloid formation by WT and Δ51-91 huPrP23-144 bound to varying amounts of Cu^2+^ (filled circles; see inset) and by WT huPrP23-144 bound to one molar equivalent of Cu^2+^ and seeded with 2% (w/w) of pre-formed WT huPrP23-144 amyloid (open circles) monitored by ThT fluorescence. (b) and (c) Representative AFM images and height profiles for amyloid fibrils formed by (b) WT and (c) Δ51-91 huPrP23-144 bound to one molar equivalent of Cu^2+^.

Next, huPrP23-144 at concentration of 400 μM bound to varying amounts of Cu^2+^ was converted to amyloid fibrils in autocatalytic manner by addition of potassium phosphate buffer, and the kinetics of fibril formation were monitored by the ThT fluorescence assay (Fig. 2a).^42^ In the absence of Cu^2+^, both WT and Δ51-91 huPrP23-144 amyloids formed with a lag phase of ∼3–4 hours, in agreement with earlier studies.^30,43^ In the presence of one molar equivalent of bound Cu^2+^, fibril formation by both proteins was characterized by a relatively small increase in the lag phase to ∼4–5 hours. Binding of increasing amounts of Cu^2+^, up to four molar equivalents, to huPrP23-144 resulted in further modest lengthening of the lag phase for spontaneous (*i.e.*, non-seeded) huPrP23-144 fibrillization reaction. The finding that with one equivalent of bound Cu^2+^ the lag phase to amyloid formation for Δ51-91 huPrP23-144 is longer than that for WT huPrP23-144 may be associated with somewhat different conformations adopted by these nominally dynamically disordered proteins upon Cu^2+^ binding, albeit no high-resolution data are available to substantiate this. Furthermore, as expected, in the presence of pre-formed WT huPrP23-144 fibril seeds the lag phase of fibril formation for Cu^2+^-bound huPrP23-144 was completely eliminated.

Morphologies of the amyloid fibril samples used for solid-state NMR studies were routinely assessed by using AFM. As shown in Fig. 2b and 2c, respectively, fibrils formed by WT and Δ51-91 huPrP23-144 containing one molar equivalent of bound Cu^2+^ have a uniform long, thread-like appearance that resembles that of corresponding fibrils obtained in the absence of bound Cu^2+^. On the other hand, the presence of bound Cu^2+^ appears to somewhat alter the fibril morphology resulting in smoother, less twisted fibrils as illustrated by the corresponding fibril height profiles.

### 2.3. Solid-state NMR studies of Cu^2+^-bound huPrP23-144 amyloids

In order to further verify that amyloid fibrils used for solid-state NMR studies indeed contain bound Cu^2+^ ions after multiple cycles of washing with 50 mM phosphate buffer at pH 6.4 prepared in absence of CuCl_2_ using ultrapure bioreagent grade (>99.0%) potassium phosphate salts and corresponding to the fibrillization conditions, we carried out standard inversion-recovery measurements of bulk amide ^1^H longitudinal relaxation times (T_1_) (Fig. S1, ESI†). These measurements show a significant ∼3–4-fold reduction in amide ^1^H T_1_ for fibrils formed by WT and Δ51-91 huPrP23-144 bound to one molar equivalent of Cu^2+^ relative to the corresponding fibrils generated in the absence of bound Cu^2+^ ions, in line with results obtained for microcrystalline proteins containing covalent Cys-EDTA-Cu^2+^ side-chains.^44,45^ For fibrils formed by huPrP23-144 containing two molar equivalents of bound Cu^2+^, an additional ∼3-fold reduction in the amide ^1^H T_1_ was observed.

**Fig. 3:**
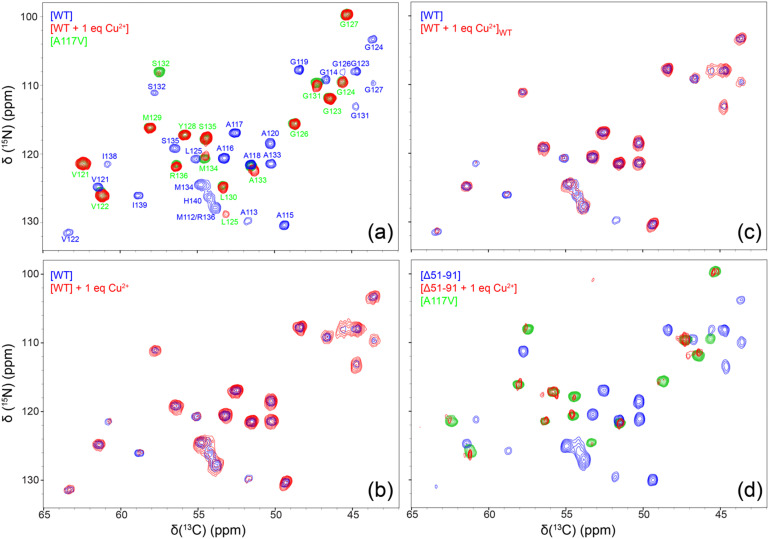
2D ^15^N–^13^Cα solid-state NMR chemical shift correlation spectra (red contours) of amyloid fibrils formed by (a) WT huPrP23-144 containing one molar equivalent of bound Cu^2+^, (b) WT huPrP23-144 incubated with one molar equivalent of Cu^2+^ following fibril formation, (c) WT huPrP23-144 containing one molar equivalent of bound Cu^2+^ seeded with 2% (w/w) pre-formed WT huPrP23-144 fibrils, and (d) Δ51-91 huPrP23-144 containing one molar equivalent of bound Cu^2+^. As appropriate, the spectra are overlaid with reference spectra corresponding to WT [panels (a)–(c)] or Δ51-91 huPrP23-144 [panel (d)] fibrils (blue contours) and huPrP23-144 A117V fibrils (green contours) with resonance assignments shown in panel (a). All spectra were recorded at 800 MHz, 11.111 kHz MAS rate and effective sample temperature of *ca.* 5 °C.

To gain residue-specific insight into the effect of bound Cu^2+^ ions on the molecular conformation and the degree of order within the core region of huPrP23-144 fibrils, we recorded fingerprint 2D ^15^N–^13^Cα chemical shift correlation solid-state NMR spectra for these fibrils generated in the absence of Cu^2+^ or in the presence of one molar equivalent of bound Cu^2+^. These spectra, shown in Fig. 3a, reveal that fibrils formed by Cu^2+^-bound huPrP23-144 adopt a core conformation that is highly ordered at the molecular level, but clearly distinct from that of WT huPrP23-144 amyloid. Remarkably, spectra of the fibrils formed by Cu^2+^-bound huPrP23-144 were found—in a consistent and reproducible manner (Fig. S2, ESI†)—to be very similar to those reported previously by our group for fibrils formed by the A117V mutant of huPrP23-144^27,34^ as well as several other huPrP23-144 single residue core mutants.^33^ The huPrP23-144 A117V amyloid core was previously found to map to residues ∼121–138 (*vs.* ∼112–141 for WT huPrP23-144 fibrils) in a mostly β-strand conformation, and appears to correspond to one particularly stable fold that is accessible to PrP23-144 within its amyloid structural landscape.^27,34^ Additionally, we found that huPrP23-144 bound to two molar equivalents of Cu^2+^ also adopts the A117V-like amyloid core structure (Fig. S3, ESI†); based on this finding solid-state NMR studies of amyloids formed by huPrP23-144 containing more than two equivalents of bound Cu^2+^ were not pursued. As additional controls, we also recorded 2D ^15^N–^13^Cα solid-state NMR spectra of huPrP23-144 fibrils with one molar equivalent of Cu^2+^ added to the sample post fibril formation (Fig. 3b), and fibrils formed by huPrP23-144 containing one equivalent of bound Cu^2+^ seeded with pre-formed WT huPrP23-144 amyloid (Fig. 3c). In both cases, fibrils clearly exhibit the WT huPrP23-144 amyloid core structure with concomitant reduction of cross-peak intensity for residues A113 and I138-H140 likely caused by backbone ^1^H, ^13^C and ^15^N transverse paramagnetic relaxation enhancements^41,46^ due to Cu^2+^ binding at H111 and H140 (as well as H96 and other more distant sites). Collectively, these solid-state NMR data indicate that, in the absence of seeding with pre-formed WT huPrP23-144 amyloid, Cu^2+^ binding to multiple sites in monomeric huPrP23-144 (including the octapeptide repeats and H96, H111 and H140) leads to huPrP23-144 adopting an alternate stable A117V-like core fold that is distinct from the WT fold. Given that, akin to the Ala to Val mutation at position 117 in huPrP23-144,^47^ the binding of one molar equivalent of Cu^2+^ to huPrP23-144 has a relatively minor effect on the fibrillization kinetics (*cf.*, Fig. 2a), adoption of this A117V-like core structure by Cu^2+^-bound huPrP23-144 may potentially be a consequence of a certain degree of structuring of the dynamically disordered huPrP23-144 by the bound Cu^2+^ ions.^10–14^

To evaluate the relative importance of individual Cu^2+^ binding sites for the apparent conformational strain switching from WT to A117V-like core fold upon Cu^2+^ binding to the monomeric huPrP23-144 prior to amyloid formation, we extended our solid-state NMR studies above to include the Δ51-91 huPrP23-144 deletion variant. The latter variant, which was previously found to adopt the WT huPrP23-144 amyloid core fold,^30^ is missing the entire octarepeat region and, thus, able to bind Cu^2+^ ions through H96, H111 and H140. As shown In Fig. 3d, the 2D ^15^N–^13^Cα solid-state NMR spectra of fibrils formed by Δ51-91 huPrP23-144 with one molar equivalent of bound Cu^2+^ are also indicative of the A117V-like core structure, in analogy to Cu^2+^-bound WT huPrP23-144—note that the reduction in overall spectral intensity for fibrils of Δ51-91 huPrP23-144 with one molar equivalent of bound Cu^2+^ relative to the corresponding fibrils not containing bound Cu^2+^ ions is related to a limited amount of sample present in the solid-state NMR rotor, and that the variations in peak intensities for individual residues in WT and Δ51-91 huPrP23-144 fibrils are due to μs–ms protein backbone dynamics in the fibril core.^26,28^ This finding suggests that the binding of Cu^2+^ ions to three histidine residues, H96, H111, H140 (which are located outside the octarepeat region in WT huPrP23-144), is the primary driver of the observed fibril conformational change.

## 3. Concluding remarks

Previous studies of amyloids formed by several PrP23-144 variants have revealed that fibrils consist of a ∼20–30 residue C-terminal β-core preceded by an extended dynamically disordered N-terminal domain.^27,31–34^ Furthermore, these studies showed that relatively conservative mutations or deletions of core residues can lead to the formation of structurally distinct amyloid strains. In the present study, we investigated the binding of Cu^2+^ to monomeric huPrP23-144 and its impact on amyloid fibril assembly and conformation, using a combination of solution and solid-state NMR spectroscopy as the primary tools. The most remarkable finding of our study is that the binding of a single molar equivalent of Cu^2+^ to monomeric huPrP23-144 greatly impacts its fibrillization, leading to formation of a structurally distinct amyloid strain that resembles that previously found for fibrils formed by A117V huPrP23-144 and several other huPrP23-144 single residue core mutants.^27,33,34^ Additionally, we found that upon seeding with pre-formed WT huPrP23-144 amyloid Cu^2+^-bound huPrP23-144 adopts a fibril structure resembling that of WT huPrP23-144, but not A117V huPrP23-144. The latter observation is suggestive of efficient conformational adaptation and templating of the Cu^2+^-bound huPrP23-144 monomers onto the WT huPrP23-144 fibril scaffold.

Since huPrP23-144 contains multiple Cu^2+^ binding sites, including the tandem PHGGGWGQ octapeptide repeats spanning residues 60–91 and histidines 96, 111 and 140, analogous studies were performed for the Δ51-91 deletion mutant of huPrP23-144 that, despite lacking the entire octarepeat region, adopts a WT amyloid core fold.^30^ Akin to WT huPrP23-144, the latter variant in the presence of one molar equivalent of bound Cu^2+^ was also found to form fibrils with a A117V-like structure, suggesting that Cu^2+^ binding to histidine residues 96, 111 and 140 is sufficient to induce the observed conformational change, possibly due to the bound Cu^2+^ resulting in a partial structuring of the intrinsically disordered monomeric huPrP23-144^10–14^*en route* to amyloid assembly.

## 4. Materials and methods

### 4.1. Protein expression and purification

Plasmid encoding for huPrP(23-144) with a N-terminal His_6_-tag and thrombin cleavage site has been described previously.^43^ As described in previous studies,^27,30^ uniformly ^13^C,^15^N-labeled protein was expressed in *E. coli* (BL21 DE3) in minimal media containing ^13^C-glucose and ^15^NH_4_Cl as the sole carbon and nitrogen sources, respectively. Protein was purified by Ni-NTA affinity chromatography, and the His_6_-tag cleaved using biotinylated thrombin. Thrombin and residual His_6_-tag were removed using streptavidin-agarose beads and dialysis against ultrapure water, respectively. Protein purity was verified by MALDI mass spectrometry and SDS-PAGE.

### 4.2. Preparation of amyloid fibrils for solid-state NMR spectroscopy

Solutions of lyophilized huPrP23-144 at 400 μM concentration in ultrapure water were incubated at 4 °C overnight with varying amounts of Cu^2+^ added as a 400 mM aqueous solution of CuCl_2_. Fibril formation was initiated by addition of 1 M potassium phosphate pH 6.4 buffer to a final potassium phosphate concentration of 50 mM at pH 6.4. Amyloid fibrils were formed under quiescent conditions at 25 °C as described previously.^32–34^

### 4.3. Thioflavin T fluorescence and atomic force microscopy

Kinetics of huPrP23-144 fibril formation in the absence and presence of Cu^2+^ were monitored by using the ThT fluorescence assay.^42^ The morphologies of fibril samples used for solid-state NMR analysis were evaluated by atomic force microscopy as described in detail in previous studies.^30,34^

### 4.4. NMR spectroscopy

Solution NMR ^15^N–^1^H HSQC spectra for huPrP23-144 dissolved at a concentration of 400 μM in aqueous MES pH 6.4 buffer containing varying amounts of Cu^2+^ (note that MES was used for these experiments instead of phosphate buffer to avoid protein aggregation/fibril formation) were recorded at 25 °C using an 850 MHz Bruker Avance III HD spectrometer equipped with a TCI CryoProbe. Amyloid fibril samples for solid-state NMR studies were incubated for 48 h at 25 °C and centrifuged. Pellets were washed three times with 50 mM potassium phosphate pH 6.4 buffer and packed into Bruker 3.2 mm zirconia rotors. Solid-state NMR ^15^N–^13^Cα chemical shift correlation spectra were recorded on an 800 MHz Bruker Avance III HD spectrometer equipped with a 3.2 mm triple-resonance ^1^H–^13^C–^15^N E^free^ probe. MAS frequency and effective sample temperature were maintained at 11.111 kHz and *ca.* 5 °C, respectively, and other experimental parameters were similar to those described in previous studies.^30,32^ Spectra were processed and analyzed using nmrPipe^48^ and Sparky.^49^

## Author contributions

CPJ designed the experiments. VS, TG and ZS prepared samples. VS and DC performed experiments. VS analyzed data and prepared figures. VS, WKS and CPJ wrote the manuscript.

## Data availability

The data supporting this article have been included as part of the manuscript and ESI.†

## Conflicts of interest

There are no conflicts of interest to declare.

## Supplementary Material

CP-026-D4CP03593C-s001
