# Supplementary material for: Copper binding alters the core structure of amyloid fibrils formed by Y145Stop human prion protein
Source: Phys Chem Chem Phys. 2024 Oct 8;26(41):26489–96. doi: 10.1039/d4cp03593c (PMC11469299; doi:10.1039/d4cp03593c)
Supplement: CP-026-D4CP03593C-s001 [file CP-026-D4CP03593C-s001.pdf]

Supplementary Information (SI) for Physical Chemistry Chemical Physics.
This journal is © the Owner Societies 2024

Supplementary Information

Copper binding alters the core structure of amyloid fibrils formed by Y145Stop human prion protein

Vidhyalakshmi Sridharan,[a] Tara George,[a] Daniel W. Conroy,[a] Zach Shaffer,[a] Witold K. Surewicz[b] and Christopher P. Jaroniec[a]*

[a] *Department of Chemistry and Biochemistry, The Ohio State University, Columbus, OH, United States*
[b] *Department of Physiology and Biophysics, Case Western Reserve University, Cleveland, OH, United States*
*E-mail: jaroniec.1@osu.edu

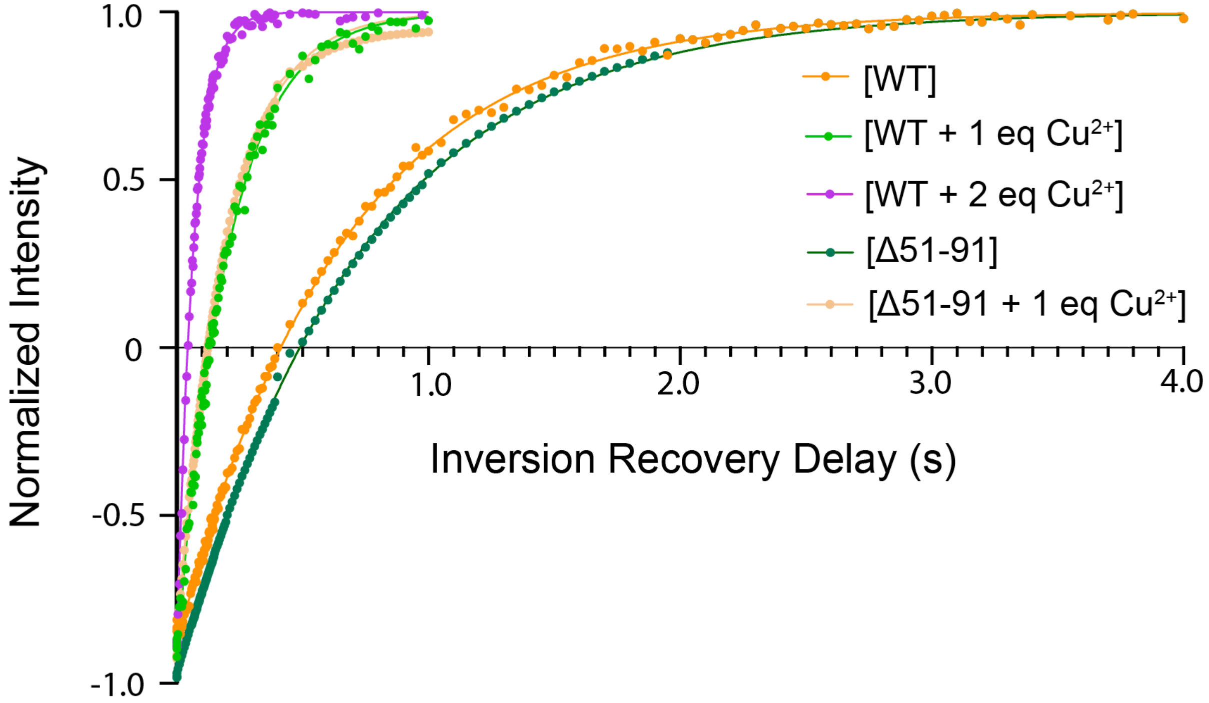

Fig. S1. Measurements of bulk amide ^1H T_1 relaxation time constants for amyloid fibrils formed by WT and Δ51-91 huPrP23-144 containing different amounts of bound Cu^{2+} as indicated in the inset. The ^1H T_1 values extracted from the data were as follows, WT: 652 ± 6 ms, WT + 1 eq Cu^{2+}: 204 ± 4 ms, WT + 2 eq Cu^{2+}: 66 ± 1 ms, Δ51-91: 760 ± 4 ms, Δ51-91 + 1 eq Cu^{2+}: 185 ± 3 ms.

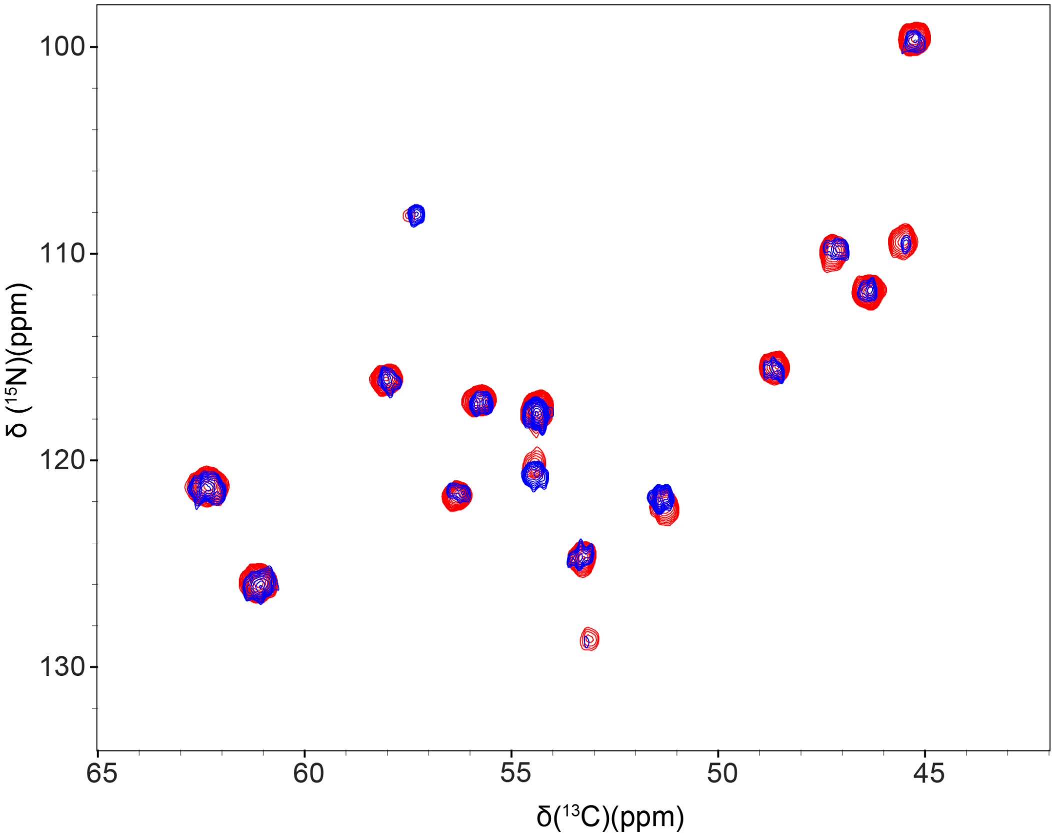

Fig. S2. Comparison of 2D ^{15}N-$^{13}C\alpha$ solid-state NMR spectra recorded for two independent preparations of amyloid fibrils formed by huPrP23-144 containing one molar equivalent of bound Cu^{2+}, illustrating the high degree of sample reproducibility. For experimental parameters see main text and Fig. 3 caption.

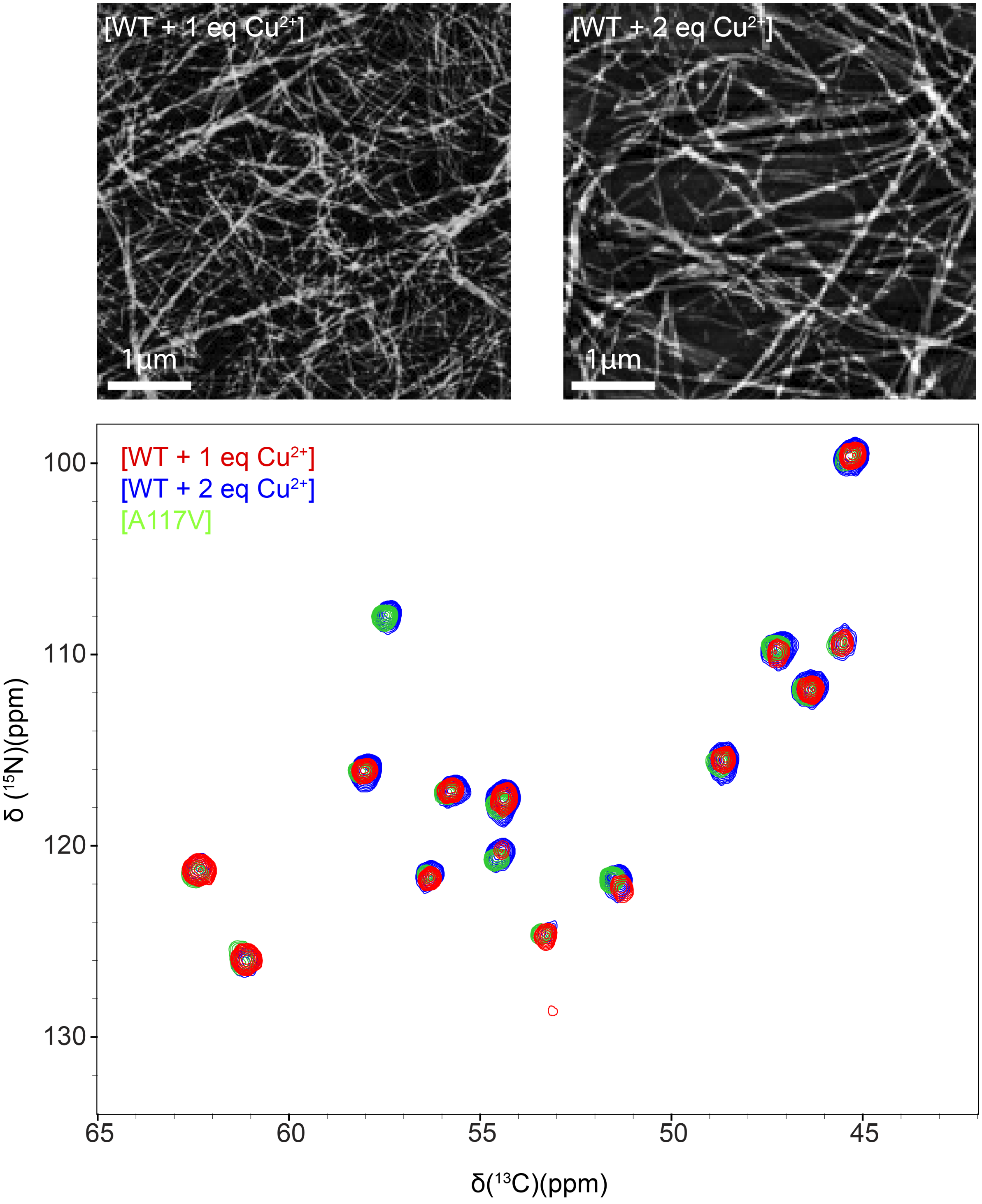

Fig. S3. AFM images (top) and 2D ^{15}N-$^{13}C\alpha$ solid-state NMR spectra (bottom) of fibrils formed by huPrP23-144 containing two molar equivalents of bound Cu^{2+} (blue contours). The NMR spectra are overlaid with corresponding spectra of fibrils formed by huPrP23-144 containing one equivalent of bound Cu^{2+} (red contours) and A117V mutant of huPrP23-144 (green contours). For experimental parameters see main text and Fig. 3 caption.
